# Case of Placenta Prævia

**Published:** 1846-09

**Authors:** G. F. Stickings


*Case of Placenta Prævia.* By G. F. Stickings, Esq. On the 20th instant, at 1 A. M., I was requested to attend Jane Bolton, aged 40, in labor of her twelfth child. On my arrival I was informed that she had had flooding for upwards of three hours: it had much increased during the last hour, and was still continuing. That she had lost much blood was evident from the bedding being completely saturated. On making an examination per vaginam I found the os uteri considerably dilated; the placenta presenting, and attached around its cervix. Having on a former occasion seen the value of removing the placenta, when so presenting, previously to the birth of the child, I hesitated not a moment (especially as the parturient process was active), but proceeded to separate it carefully from its attachment. This was accomplished without much difficulty, the hemorrhage ceasing immediately on its removal. The head presented naturally; the uterus acted with vigor, and a living male child was expelled. I am happy to state that the patient is doing extremely well.

I offer this as an additional proof of the value of the new mode of practice, in cases of presentation of the placenta.—*Med. Gaz.*

